# Strategies to Improve Health Communication: Can Health Professionals Be Heroes?

**DOI:** 10.3390/nu12061861

**Published:** 2020-06-22

**Authors:** Eva L. Jenkins, Jasmina Ilicic, Annika Molenaar, Shinyi Chin, Tracy A. McCaffrey

**Affiliations:** 1 Department of Nutrition, Dietetics and Food, Monash University, Notting Hill 3168, Australia; eva.jenkins@monash.edu (E.L.J.); annika.molenaar@monash.edu (A.M.); 2 Monash Business School, Monash University, Caulfield East 3145, Australia; jasmina.ilicic@monash.edu; 3 School of Media and Communications, Royal Melbourne Institute of Technology, Melbourne 3004, Australia; shinyi.chin@rmit.edu.au

**Keywords:** social media, social media influencers, nutrition professionals, trustworthiness, authenticity, emotional message appeals, university students, young adults

## Abstract

Communicating evidence-based nutrition messages to the public is challenging and is often in conflict with popular opinions, particularly from social media influencers (SMIs). In order to increase engagement with nutrition professionals (NPs) on social media, we aimed to explore young adults’ perceptions of the authenticity and trustworthiness of SMIs and NPs Instagram posts. A cross-sectional questionnaire was administered to students (*n* = 149) from an Australian University. Participants viewed a real-life Instagram profile and one post from both a NP and a SMI. Main outcomes were post authenticity and trustworthiness, and emotional message appeals measured on five-point Likert scales. Regression models were developed to assess whose post (the NP or SMI) was perceived to be more authentic and trustworthy. Participants were young adults (median age (25th, 75th percentiles): 20 (19,21)), with approximately half identifying as female. A high heroic message appeal (+1SD above mean) significantly increased the perceived authenticity of the NPs post only (*p* = 0.01). Post authenticity enhanced post trustworthiness, but only when a heroic message appeal was used by the NP. When appropriate, NPs should convey positive emotions such as bravery and success to enhance the authenticity and trustworthiness of their posts.

## 1. Introduction

Communicating health messages clearly and openly to the public is challenging, as much of the evidence focuses on longevity and disease prevention, rather than behaviours that generate instant results and gratification [1]. Previous research has indicated that young adults and university students are often uninterested in the long-term benefits or consequences of their current eating behaviours [2,3]. Whilst nutrition science has enabled countless discoveries and progressions in science [4], nutrition science is more complicated than other disciplines of science in multiple ways. Firstly, food is an essential part of every human’s life, thus many people have a vested interest in nutrition and care about their health [5]. Diet quality is typically poor, particularly amongst young adults and university students, and the current obesogenic climate makes healthy choices more challenging than ever before [6,7].

Secondly, with the widespread use of social media (SM; see Appendix A for a glossary of terms), many people without formal qualifications, such as celebrities and social media influencers (herein referred to collectively as SMIs; Appendix A) are sharing science-related information that is influential and highly accessible to a wide audience. The discipline of nutrition science is riddled with questions surrounding authenticity, trustworthiness, and credibility [1,8]. The oversimplification of translating study findings by some media outlets causes confusion among the public. These translation activities often ignore key differences in study design—i.e., methods and the population of interest (human vs. animal studies)—thus causing confusion and lack of trust when results are conflicting. Our work has shown that government translation of nutrition research (i.e., the Australian Guide to Healthy Eating) fails to capture the attention of young adults as it is not relevant and applicable to their lives, unlike content from SMIs [2].

SM has enhanced the proliferation of ‘fad diets’, particularly those which restrict whole food groups (e.g., the paleo diet; grains and dairy), thus limiting the variety in our diets. Young adults are the biggest consumers of SM content, with approximately 70% of 18–24-year-olds using Instagram (Appendix A) [9], and University students feeling permanently connected to SM [10]. However, our systematic review identified that the use of SM for health interventions in young adults had limited success with highly variable engagement rates (Appendix A) ranging from 3–69% [11]. Health professionals often have jobs outside of SM, and are bound by professionalism principles, [12] and thus may not be as candid or have as much time as SMIs to grow their audience and spread evidence-based information, potentially limiting their ability to engage young people.

In the existing post-truth era (Appendix A), experts are often less highly regarded by young adults and emotional message appeals (Appendix A) are typically the most effective methods of communication [13,14,15]. Previous research has detailed the effectiveness of positive emotional message appeals, such as humour (Appendix A), as a way to increase engagement on SM [16,17,18]. On Instagram, young adults are surrounded by the pressure of idyllic lifestyles, frequently being exposed to accounts promoting ‘#fitspiration’ and ‘#cleaneating’ encouraging self-comparison and negative self-image [19,20]. Many of the photos on Instagram are heavily edited, creating unrealistic expectations and impacting vulnerable people; particularly women, who are trying to fit-in with others online [20,21]. Exposure to image-related content is associated with higher body dissatisfaction, dieting or restricting food, overeating, and choosing healthy foods in young adults; hence, the mental and emotional impact of SMa differs between individuals [20]. Young adults often feel pressure to be healthy due to the tendency to compare themselves to others, however, they frequently lack the motivation or ability to successfully make healthy behaviour changes due to many environmental and social barriers [2]. This sub-population is an important target for health interventions to increase the capacity to adopt healthy eating behaviours, which aid in reducing the risk of chronic disease later in life [2,22,23]. Therefore, exploring the perceived trustworthiness, authenticity, and message appeals of nutrition professionals (NPs; Appendix A) and SMIs on Instagram could be useful to inform health communication techniques targeted at young adults in the future. Some NPs could be considered SMIs, but the distinguishing factor in our study is the presence or absence of a tertiary qualification in nutrition (Appendix A).

This paper is informed by the self-determination theory (Appendix A) and the source credibility model (Appendix A). The self-determination theory encompasses the concept of authenticity, defined as “being true to the self in terms of an individual’s thoughts, feelings, and behaviours reflecting their true identity” (Appendix A) [24]. In the marketing literature, individuals tend to perceive another person (e.g., a celebrity) as authentic when the other person’s actions reflect their autonomous, self-determining, true self [25]. Those who are perceived as authentic have a higher level of influence over others, both online and offline [26]. Source credibility is a communicator’s positive characteristics that affect the receiver’s acceptance of a message, encompassing attractiveness, trustworthiness, and expertise [27]. In these analyses, we focus primarily on trustworthiness.

Our scoping review has highlighted the paucity of research from health and nutrition science that considers the trustworthiness or authenticity of a spokesperson’s communications [28]. The aim of this paper was to understand the differences in consumer’s perceptions of NPs and SMIs on Instagram through exploring authenticity and trustworthiness. In marketing, authenticity and trustworthiness are distinct constructs; however, research has found that the perceived authenticity of celebrities and brands increases their trustworthiness (e.g., authentic brands are more likely to be trusted by consumers compared to inauthentic brands) [29,30]. Based on the health and marketing literature, it is hypothesised that SMIs are similar to celebrity endorsers (who are perceived as authentic [31]; Appendix A) as they are both recognised as authorities in their specific fields [29], and, therefore, consumers will perceive the Instagram post of the SMI (versus NP) as more: (1) authentic; and (2) trustworthy. Given that positive or gain-framed messages have been found to more effectively promote the adoption of healthy eating behaviours [2,32,33], we hypothesise that a positive message appeal will influence the authenticity of SMIs’ (versus NPs) Instagram posts.

## 2. Materials and Methods

### 2.1. Participants and Recruitment

A cross-sectional pilot study was used to explore University students’ perceptions of a SMI and NP. Prior to recruitment, this study was approved by the Monash University Human Research Ethics Committee (approval number: 19201). Participants were a convenience sample of marketing research methods students enrolled in a Bachelor of Business at a Metropolitan University in Australia. Recruitment occurred via Sona, an online research portal in which University researchers advertise current research opportunities for students to participate in. Only second-year students enrolled in the marketing research methods subject could participate. Within this sample, there were no specific inclusion or exclusion criteria. A brief description of the study was provided on the Sona portal, with 13 possible time slots for participation available, allowing a maximum of 220 participants. Participation in the study contributed to 3% of the students’ coursework requirements. Overall, 152 participants attended the sessions.

### 2.2. Procedure

Data collection occurred over two days on 13 May and 16 May 2019. The study was held in a Behavioural Laboratory at the University. Participants attended one 30-min session of their choice, occurring between 9:00 a.m. and 5:00 p.m. on either of the days of data collection. On arrival, the participants entered the laboratory and were given a unique participant identification number to access the questionnaire. Participants’ names and identification numbers were kept confidential at all times. Participants sat down at a desk of their choice where a laptop was set up to begin the questionnaire. Informed consent was required before commencing, or alternatively, if the participant did not consent, they could leave without participating. There were barriers around individual desks to ensure the privacy of participants, and headphones were supplied to reduce noise disturbance.

### 2.3. Pilot Questionnaire

#### 2.3.1. Development

The pilot questionnaire was online, self-administered, and generated on Qualtrics^®^ (Provo, UT, USA) software. The pilot questionnaire was developed through collaboration with researchers from the nutrition science (ELJ, AM, TMC) and marketing disciplines (JI, SC) and informed by our prior research [2,11,17,18,20,25,34,35]. A template was developed from a previous questionnaire [36] conducted as part of the *Communicating Health* study [34], then further refined to be specific to this study. The questionnaire was piloted to assess the format, layout, and length, with the feedback used to further refine the questionnaire before completing the final draft. The final questionnaire consisted of 75 questions covering 14 topics Supplementary (1). Originally, the questionnaire had a broad scope and included questions relating to communication objectives, SM behaviour, and physical activity. However, based on the findings of our scoping review [28] these analyses focus only on the results relating to trustworthiness, authenticity, and message appeals [16,37,38].

#### 2.3.2. Stimulus

The participants were shown screenshots of real-life Instagram posts from a SMI and a NP. These posts were sourced from Socialbakers^©^ (Prague, Czech Republic), an online SM analytical company, as part of a previous student project [17]. Previously, the top 10 most popular Facebook profiles among Australian users for both lifestyle SMIs and NPs were identified (methods detailed elsewhere) [17]. The highest performing accounts with Instagram (based on the number of followers) from both categories were chosen to be included in this questionnaire. The accounts identified were both female, with a differing number of followers at the time of data collection (NP = 91.2 thousand; SMI = 11.3 million). Over the period of 25 June 2018 to 29 July 2018, Socialbakers^©^ was used to determine the highest performing post (based on engagement i.e., sum of likes and comments) on each of the Instagram accounts. These posts (*n* = 2) were then included in the pilot questionnaire. Photos were edited to ensure consistency across the profiles (i.e., removing comments whilst leaving the number of likes visible). There was a difference in the number of likes between sources (NP 2686 likes, SMI 282, 711 likes). For this pilot questionnaire, a screenshot of the Instagram profile (small profile picture and biography) for both the NP and SMI, and their corresponding post (highest performing) was also included. The NP’s profile biography stated that she was a dietitian, body positive, and an author of a nutrition-related book, and her post included in the questionnaire discussed body image and body positivity Supplementary (1). The SMI’s profile biography included her relationship status, her pregnancy progress, and advertising for her fitness program, and her post discussed her long-term relationship. The order in which participants saw the NP or SMI was randomised (by Qualtrics^®^ online software) to minimise confounding order-effects.

#### 2.3.3. Measures

Familiarity and likeability of the source was first measured based on the Instagram profile (measured using a validated semantic differential scale, each with five items) [39]. The main outcomes were post trustworthiness [27] and post authenticity [31] assessed using validated scales, as well as perceived message appeal used in the Instagram post (measured by eight individual items on a five-point Likert scale ranging from ‘strongly disagree’ to ‘strongly agree’; affiliation, hope, humour, heroic, convenience, guilt, sorrow, fear) [16]. Participants answered questions related to their own nutrition knowledge, healthy eating behaviours, and quality of life, also assessed via statements on a five-point Likert scale [40,41,42,43]. All scales had been previously used in the literature. Participant’s demographic data such as gender, age, country of birth, employment status, and self-reported weight and height were also collected.

### 2.4. Statistical Analysis

#### 2.4.1. Software

Questionnaire data was collected in and exported from Qualtrics^®^ online software into IBM SPSS^®^ (Statistical Package for Social Sciences; Version 26, Armonk, NY, USA) for Windows. The PROCESS macro was used for regression analysis [44].

#### 2.4.2. Statistical Tests

Participants who did not respond to demographic questions were excluded from analysis (*n* = 3). General data cleaning took place prior to analysing data. Variables that were negatively worded within a scale were reverse coded. To assess the reliability of the scales used and ensure the items in each scale were measuring the same concept, Cronbach’s alpha (α) was calculated. If the α value was above 0.7 the scale was considered a reliable measure. Once reliability was confirmed, an average score for each subscale was calculated by dividing the total scale score by the number of items within the scale (e.g., trustworthiness, five items). For any scales where α was below 0.7 (i.e., nutrition knowledge), factor analysis was conducted, and the scale was split into the appropriate number of sub-scales with an α value above 0.7.

The body mass index (BMI) (kg/m^2^) of participants was calculated using self-reported weight and height data from the questionnaire. BMI (kg/m^2^) was classified into three groups: underweight (<18.49 kg/m^2^), healthy weight (18.5–24.99 kg/m^2^), and overweight or obese (>25 kg/m^2^), based on the World Health Organisation (WHO) cut-offs [45].

**Figure 1:**
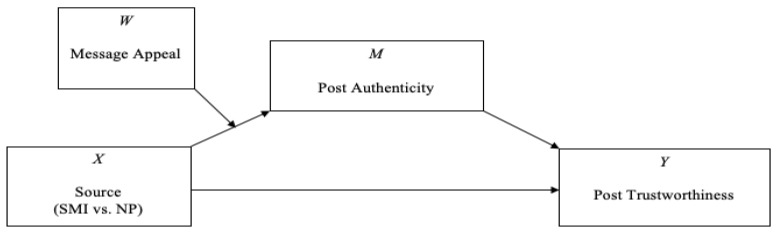
Illustration of PROCESS Model 7—Moderated Mediation Model (with hypothesised analysis). SMI, social media influencer; NP, nutrition professional. Letters (*M*, *W*, *X*, *Y*) are variables not abbreviations.

The PROCESS macro bootstrapping procedure (*n* = 10,000) was used to test for moderated mediation (PROCESS Model 7; See Figure 1) [44]. The PROCESS macro tests the effect of the interaction (i.e., source (SMI/NP) × message appeal) on the mediator (i.e., post authenticity), as well as the effect of the mediator (i.e., post authenticity) on the outcome variable (i.e., post trustworthiness). The independent variable was dummy coded to reflect the source of the profile (i.e., SMI = 0, NP = 1). Covariates included in the regression models were participants’ gender, self-reported BMI, participants’ subjective nutrition knowledge, subjective nutrition expertise, participants’ healthy eating behaviour, self-reported quality of life, and the perceived familiarity and likeability of the source.

## 3. Results

### 3.1. Demographics

A total of 149 (97.4%) participants with a median age of 20 years (19, 21, values are 25th and 75th percentiles) completed the survey. Approximately half of participants identified as female (54.4%) and were born in Australia (51.7%; Table 1). Males were taller and heavier, and consequently had a greater BMI (kg/m^2^) than females (*p* = 0.002). However, there was no difference in the proportion of participants between each BMI category (Table 1). The majority of participants studied full time (98%) and engaged in part-time or casual work (62.1%; Table 1). Self-reported quality of life was generally high (Table 1). Participants believed they had high levels of nutrition knowledge, with females rating significantly higher than males (*p* = 0.026; Table 1).

### 3.2. Reliability of Scales

Cronbach’s α was above 0.7 for all scales including post trustworthiness (5 items), post authenticity (4 items), source familiarity (4 items), source likeability (3 items), quality of life (4 items), healthy eating behaviours (3 items), and nutrition knowledge (split into general nutrition knowledge: 4 items, and nutrition expertise: 2 items), allowing the mean scores to be used for further analysis (Table 2).

### 3.3. Descriptive Statistics

The NP was perceived as significantly more authentic and trustworthy than the SMI (Table 3). In terms of perceived message appeal, the NP was more likely to use a heroic message appeal, with the SMI using affiliation (feelings of love, belonging, and togetherness) and guilt/shame message appeals (*p* < 0.001; Table 3).

### 3.4. Regression Results

Separate models estimated the effect of the interaction between the source and each of the eight message appeals on the dependent variable: post authenticity (Table 4). Each of the separate models included participants’ gender, self-reported BMI, source familiarity, source likeability, self-reported quality of life, participants’ healthy eating behaviour, participants’ subjective nutrition knowledge, and subjective nutrition expertise. The results showed that the perceived heroic appeal (conveys bravery, nobility, and admiration; Appendix A) communicated by the source was the only predictor of post authenticity in this case. As such, we further explore this relationship in greater detail below, while also including post trustworthiness in our model.

**Figure 2:**
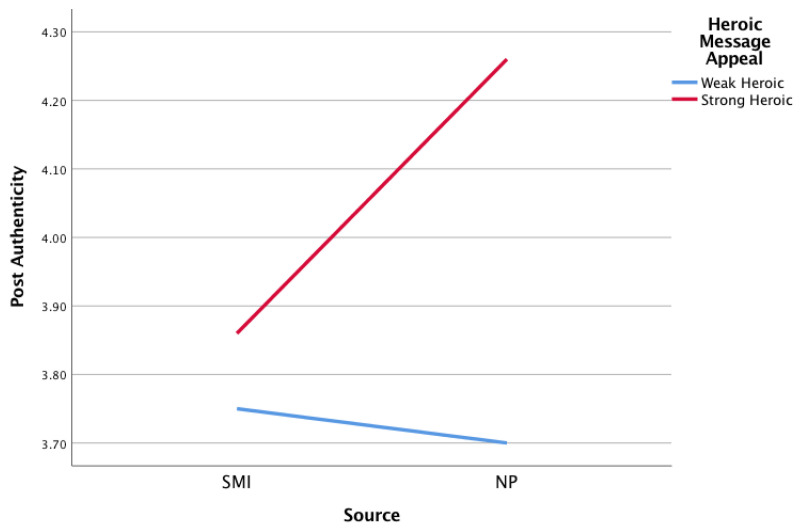
Illustration of the interaction effect (Source × Heroic Message Appeal) on post authenticity.

Model 1 (Table 5) estimated the effect of the interaction between the source and heroic message appeal on the dependent variable: post authenticity. The model included participants’ gender, self-reported BMI, source familiarity, source likeability, self-reported quality of life, participants’ healthy eating behaviour, subjective nutrition knowledge, and subjective nutrition expertise as covariates. The results showed that the source was a significant predictor of post authenticity. Interestingly, the NP’s post was perceived as significantly more authentic than the post by the SMI (*t* = −2.06, *p* = 0.04; M_SMI_ = 3.79, M_NP_ = 3.98). Heroic message appeal was not a significant predictor of post authenticity (*t* = −1.01, *p* = 0.31). However, the interaction (Source x Heroic Message Appeal) was found to have a significant effect on perceived post authenticity (*t* = 2.54, *p* = 0.01; Table 5, Model 1). Specifically, a strong heroic message appeal (+1 SD above the mean) significantly increased the perceived authenticity of the NP’s post only (M_SMI_ = 4.26, M_NP_ = 3.86; *t* = 2.76, *p* = 0.01; Figure 2). Both the SMI’s and NP’s posts were perceived as less authentic when their message appeal was weak in heroism (−1 SD Below the mean; M_SMI_ = 3.75, M_NP_ = 3.70; *t* = −0.39, *p* = 0.70; Figure 2). The effect of participant’s gender, source likability, and nutrition expertise on post authenticity were also statistically significant.

**Figure 3:**
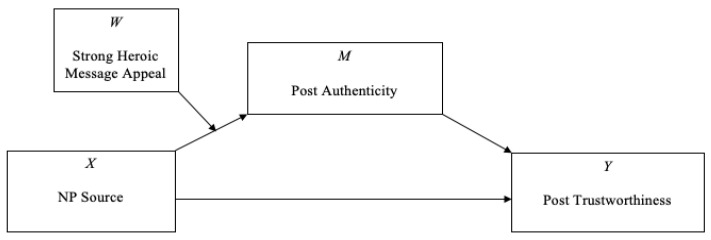
Illustration of the PROCESS Model 7. Nutrition professionals as the source of the Instagram post using a strong heroic message appeal was a significant predictor of post authenticity and subsequently, post trustworthiness. NP, nutrition professional. Letters (*M*, *W*, *X*, *Y*) are variables not abbreviations.

We then tested whether the effect of the interaction (source × heroic message appeal) on post trustworthiness was mediated by post authenticity (Table 5, Model 2). When testing for moderated mediation, the key indicator is the indirect effect of the interaction term on the dependent variable through the mediator [44]. The interaction (source × heroic message appeal) was a significant predictor of post authenticity, and post authenticity was a positive and significant predictor of post trustworthiness. As the 95% bootstrapped confidence interval for the indirect effect of the interaction on post trustworthiness through post authenticity did not include zero (*β* = 0.15, 95% CI = 0.02 to 0.29), a significant moderated mediation effect was demonstrated. Specifically, the results provide evidence that post authenticity enhances post trustworthiness only when participants perceive a strong heroic message appeal being used by a NP (Figure 3). The effect of participants’ gender and participants’ nutrition expertise on post trustworthiness was also statistically significant.

## 4. Discussion

It was hypothesised that the SMI would be more trustworthy and authentic than the NP based on the extensive celebrity endorsement literature, whereby an Instagram influencer is akin to a true celebrity [29,46]. However, the results of this study provide initial evidence that the NPs post was perceived by young adults to be more authentic, and subsequently, more trustworthy, than a SMIs post. We provide evidence of this relationship irrespective of the young adults’ gender, BMI, familiarity with the source, likability of the source, self-reported quality of life, healthy eating behaviour, subjective nutrition knowledge, or subjective nutrition expertise. Therefore, hypothesis 1 and hypothesis 2 were not supported.

Communicating health through SM is challenging, and research focused on the methodology for improving SM engagement for NPs is currently lacking. In this study, a novel concept in this field of research was examined: the perceived authenticity and trustworthiness of NPs posts compared to SMIs. SMIs often promote damaging fad-diets and share misinformation without consequence; whilst NPs promote evidence-based sustainable diet changes for disease prevention. Currently, Instagram and Facebook are unregulated in regard to health misinformation, with the exception of COVID-19 related information. In 2019, a policy was introduced that prevented diet supplements being advertised to under 18 year olds [47] which is a step in the right direction. We await the application of these techniques to other areas in regard to stemming the proliferation of misinformation. Our previous research has identified many factors that may impact the trustworthiness and authenticity of such posts such as number of followers, message appeal, and authority cues [28]. However, there is a paucity of research on the influence of either NPs or SMIs on perceived trustworthiness and authenticity of SM posts [28].

Based on our exploratory results, we further showed that the authenticity of the NP’s posts was dependent on the perceived strength of the heroic message appeal communicated. A NP’s post was perceived as more authentic, and subsequently more trustworthy, when the message appeal used in the post was perceived to be strongly heroic. On the other hand, the authenticity of the NP’s post was perceived as significantly less authentic, and subsequently less trustworthy, when their message appeal was perceived to be weak in heroism. In other words, it is suggested that, when appropriate, NPs attempt to convey positive emotions relating to heroism such as bravery, nobility, and success, in their messages in order to enhance the genuineness and realness of their posts.

Whilst the authenticity of NPs’ messages, or content, has not been directly measured to date (to the authors’ knowledge), the medical literature highlights the importance of authenticity driving the motives of health professionals, such as doctors and nurses [48]. Conceptualisations of authenticity in health surround the themes of genuineness, consistency, and caring [48,49]. Trust is also an important consideration in healthcare settings, with trusted professionals being more likely to lead their patients to better health outcomes and consequently, quality of life [50]. The current literature (from clinical settings) suggests that health professionals can develop trusting relationships through being non-judgemental and encouraging two-way interaction between themselves and patients [51]. However, young adults have previously identified lack of trust and communication difficulties as important factors contributing to negative healthcare experiences [52]. Trusting relationships are essential in achieving behavioural change over SM; as those on SM platforms who have higher trust from their audience have a higher level of influence over others’ behaviours [53]. In a review looking at the efficacy of using SM for achieving nutrition outcomes in young adults, it was found that while young adults considered SM an acceptable source for health information, they preferred a one-way conversation, regarding health with professionals through SM and did not wish to discuss their weight [11]. In addition, health and fitness information shared through University affiliated SM pages has been found to be acceptable by University students [54].

Young adults constitute the audience of many SMIs, who share their personal life online and attract a loyal following, wanting to form a personal connection. This is known as a ‘parasocial relationship’ (Appendix A), thus creating an illusion of intimacy and friendship, despite the majority of followers remaining unknown by the SMI [55]. In contrast, NPs must maintain a sense of professionalism online and, therefore, typically cannot create the same type of content without risking the loss of their professional image [56]. However, in this study, the caption in the NP’s post is a vulnerable description of when she was struggling with weight-loss. This use of vulnerability may perhaps not be the ‘norm’ for NPs on SM, but it does provide strategies for how professionalism and vulnerability may be combined, as suggested by the celebrity literature from marketing and psychology [25,57,58]. One study looking at the authenticity of bloggers found that bloggers who shared their innermost thoughts and many facets of their personal life on their blog were seen as more relatable and authentic than those who were less open about their personal life [49].

In both commercial and social marketing, message appeal is often manipulated to influence consumer’s emotions and generate a greater persuasive capacity [16]. There is limited research published in health and medicine that considers the effectiveness of varying message appeals over SM [16]. However, those published emphasise the effectiveness of positive emotional appeals to increase engagement with public health messages [59,60]. Specifically, a ‘heroic’ message appeal, associated with bravery and nobility, is not commonly researched in the literature, with many studies focusing on negative emotional appeals such as ‘guilt’, ‘fear’, and ‘shame’, or single positive appeals such as ‘humour’ [16,59]. Negative emotional appeals can often result in feelings detrimental to an individual’s wellbeing [61], and have been found to lead to the ‘flight, fight, or freeze’ response, eliciting a longer-lasting impact on the nervous system when compared to positive appeals [62]. As detailed in Self-Determination Theory, an individual should be autonomous in their decision making to attain long-lasting behaviour change [62]. Individuals exposed to negative appeals without control over their exposure (i.e., seeing an advertisement on TV), can result in an ‘avoidance motivation’, whereby an individual makes effort to avoid anything they anticipate will cause sadness and anxiety [63], hence scare tactics are not always effective in influencing behaviours. Our online conversations provide further evidence of needing to move away from negative rhetoric, as guilt appeals around healthy eating did not work on young adults who did not hold the same beliefs about health (e.g., ‘sinners’) [35]. Traditionally, health promotion organisations have maintained a serious message and utilised rational message appeals (e.g., facts, statistics) over emotional appeals, generating lower engagement rates from their audience [11,17,18,64]. Young adults have indicated that healthy eating messages would be more persuasive if they incorporated empathy, while an authoritative message was rated poorly for perceived ability to encourage healthy eating [65]. The findings from our study suggest that by focusing on positive emotional appeals such as heroism, the audience can empathetically connect with NPs, which could lead to greater engagement rates. Specifically, results from the regression analysis indicated that a perceived ‘strong’ heroic message appeal resulted in a more authentic perception of the NP’s post, and when using a ‘weak’ perceived heroic message appeal, the authenticity of both the SMI’s and NP’s post was reduced.

This research has highlighted the importance placed on message appeal in assessing the authenticity of SM posts, especially those of NPs. NPs could benefit from communicating their success and bravery to increase the authenticity of their posts. Although this study found that young adults were less likely to perceive the posts of SMIs as authentic and trustworthy, consumers are often inspired by celebrities and influencers, and follow them to learn from their experiences with different diets and exercise regimes. Many SMIs advertise one-on-one consultation sessions, and sell personalised meal plans, exercise eBooks, and nutritional supplements based on anecdotal evidence and pseudoscience [66,67,68]. As the number of followers increases for SMIs, there are more consumers trying the behaviours promoted, reflecting ‘herd behaviour’ (Appendix A), the phenomenon of individuals deciding to follow others and imitating group behaviours rather than deciding independently on the basis of their own, private information [15,69]. Herd behaviour can trigger a larger trend, which could be harmful to many people, particularly if it is based on unqualified advice. To our knowledge, there are no consistent ways for the public to identify credentialed health professionals and scientists, particularly on SM. NPs need to be cognisant of the different communication strategies used in the online environment and focus on educating the public through sharing relatable evidence-based health advice.

The profiles and posts included in the survey were sourced based on objective engagement metrics rather than being subjectively chosen by the researchers. Real-life profiles and posts were used rather than fictional characters, making the study more realistic and providing a true sense of consumers’ perceptions of the SMI and NP. Furthermore, the use of validated scales to assess consumer perceptions produced excellent Cronbach’s α scores for all measures (Table 2) and ensured the survey measured what it was intended to.

The experimental nature of this study—rather than a real-life setting, as well as the convenience sample of University students participating as part of their coursework requirements—limits the generalisability of these results to the wider population of young adults. Furthermore, the use of student participants limits the variability of results as students samples (referred to as western, educated, industrialised, rich, and democratic (WEIRD)) [70] are seen as more homogenous in terms of education level and socioeconomic status than the general public. Additionally, the two Instagram profiles sourced by Socialbakers^©^ were both young and conventionally attractive females, which could have affected the variability of results. In an effort to keep the posts as similar to real as possible, the number of likes was visible, however there was a large difference between sources (SMI 282,711, NP 2686) which could have impacted participants’ perceptions. The messages in the SMI and NP posts were also discussing different topics (NP: body image, SMI: relationships) which could have unknowingly affected results. In this pilot study, we focussed on measuring the trustworthiness of the Instagram posts. Future iterations should consider adapting the questions to measure perceived expertise and attractiveness (if the person is shown in the photo) of the SMI and NP based on the Instagram posts. Future research could use an experimental design to manipulate the message in the caption and/or number of likes on the Instagram posts to examine the effect of message topic and bandwagon cues on trustworthiness and authenticity. Further research is required to enhance the understanding of trustworthiness and authenticity on SM, as well as to validate these findings using male SMIs and NPs, and non-student young adult populations.

## 5. Conclusions

This study was the first of the authors’ knowledge to compare perceptions of SMI and NP posts on Instagram, with a focus on trustworthiness, authenticity, and message appeal. We have developed recommendations for NPs using SM (Table 6) that add to our existing recommendations from our work emanating from Communicating Health [34], particularly in relation to body image [20], nuanced messaging for groups with different attitudes and beliefs [2,35], and the need to adapt posts to the different SM platforms along with utilising strategies associated with higher engagement [17,18]. Nutrition science and health communication is fraught with difficulty in distilling complex messages into information that can be simply understood by the lay population. We found that by using a heroic message appeal, the NP post was seen as more authentic and, subsequently, more trustworthy. This research has highlighted the impact that message appeals can have on an audience, therefore posting positive, brave and successful content could be an effective way to improve the persuasiveness of health communication by NPs.

## Figures and Tables

**Table 1:** Demographic characteristics of the study population ^§.^

Measure	Overall (*n* = 149)	Male (*n* = 68)	Female (*n* = 81)
Age (years)	20.0 (19.0, 21.0)	21.0 (19.2, 22.0)	20.0 (19.0, 21.0)
Weight (kg)	64.0 (55.0, 75.0)	74.0 ^a^ (65.0, 80.0)	56.0 ^b^ (50.0, 66.5)
Height (cm)	170 (162, 177)	177 ^a^ (174, 180)	163.0 ^b^ (158, 170)
BMI (kg/m^2^)	22.2 (19.7, 25.0)	23.4 ^a^ (20.9, 25.4)	21.1 ^b^ (19.0, 23.4)
WHO cut offs for BMI [45]			
Underweight, *n* (%)	19 (12.8)	5 (7.4)	14 (17.3)
Healthy weight, *n* (%)	94 (63.1)	42 (61.8)	52 (64.2)
Overweight and Obese, *n* (%)	36 (24.2)	21 (30.9)	15 (18.5)
Country of birth			
Australia, *n* (%)	77 (51.7)	34 (50.0)	43 (53.1)
Other, *n* (%)	72 (48.3)	34 (50.0)	38 (46.9)
Asia and Pacific	65 (43.6)	30 (44.1)	35 (43.2)
Africa	2 (1.3)	2 (2.9)	0
North America	1 (0.7)	0	1 (1.2)
Europe	4 (2.7)	2 (2.9)	2 (2.5)
Employment status ^¶^			
Full-time, *n* (%)	1 (0.7)	0	1 (1.2)
Part-time or casual, *n* (%)	91 (61.1)	40 (58.8)	51 (63.0)
Not working, *n* (%)	52 (34.9)	26 (38.2	26 (32.1)
Not disclosed, *n* (%)	5 (3.3)	2 (2.9)	3 (3.7)
Study load			
Full-time, *n* (%)	146 (98.0)	66 (97.1)	80 (98.8)
Part-time, *n* (%)	3 (2.0)	2 (2.9)	1 (1.2)
Quality of life measure^‡^ [40]			
Self-reported quality of life	3.4 (3.0, 4.0)	3.4 (3.0, 4.0)	3.4 (3.0, 4.0)
Subjective nutrition knowledge ^‡^ [41]			
Self-reported general nutrition knowledge	3.4 (3.0, 4.0)	3.3 ^a^ (2.8, 4.0)	3.4 ^b^ (3.0, 4.0)
Self-reported nutrition expertise	2.5 (1.8, 3.0)	2.5 (1.5, 3.0)	2.5 (2.0, 3.0)
Healthy eating ^‡^ [43]			
Healthy eating behaviour	4.0 (3.0, 4.0)	3.83 ^a^ (2.7, 4.0)	4.0 ^b^ (3.2, 4.0)

^§^ All continuous values reported as Median (25th, 75th percentile); a and b denote significant differences between male and female median scores, using Mann–Whitney U test, *p* < 0.05; ^¶^ Significance testing unable to be calculated as expected frequency was less than 5; ^‡^ median score of a 5-point scale, minimum score 1 ‘strongly disagree’, maximum score 5 ‘strongly agree’, subjective nutrition knowledge was split into two sub-scales: self-reported general nutrition knowledge (*n* = 4 items) and self-reported nutrition expertise (*n* = 2 items). BMI, body mass index, WHO, World Health Organisation.

**Table 2:** Constructs and their associated items from scales used in the questionnaire.

Construct (Cronbach’s α) ^‖^	Measure ^¶^	Source
Scales used to assess the Instagram profiles		
Familiarity (SMI: 0.92, NP: 0.91)	I am familiar with ‘NP/SMI’	Bruner and Gordon, 2013 [39]
	I know a lot about ‘NP/SMI’	
	I follow ‘NP/SMI’ on social media	
	I read her social media posts	
Likeability (SMI: 0.88, NP: 0.86)	I find ‘NP/SMI’ likeable	Bruner and Gordon, 2013 [39]
	My impression of ‘NP/SMI’ is favourable	
	I find ‘NP/SMI’ warm and friendly	
Scales used to assess the Instagram posts		
	Assessed by asking “what emotions do you think the post is trying to show?”	
Message-appeal (measured as individual items)	Affiliation: Feelings of love, belonging, and togetherness	Yap, 2017 [16]
	Hope: Feelings of hope and reassurance	
	Humour: Feelings of fun, happiness, laughter, joy, light-heartedness, cheerfulness, comedy	
	Heroism/success: Feelings of bravery, nobility, admiration	
	Ease/convenience: Feeling that little effort is required to engage in the behaviour in the post	
	Guilt/shame: Feelings of shame and remorse	
	Sorrow: Feelings of sadness, worry, loss, misery, and unhappiness	
	Fear: Feelings of terror, dread, anxiety, panic, and alarm	
Trustworthiness (SMI: 0.89, NP: 0.91)	The content of this post seems dependable.	Adapted from Ohanian, 1990 [27]
	I find the information provided in this post reliable.	
	This post is being honest in its recommendations.	
	This post seems sincere.	
	I trust the information provided in this post.	
Authenticity (SMI: 0.88, NP: 0.93)	The content in this post is genuine.	Moulard et al., 2015 [31]
	The content in this post seems real to me.	
	The content in this post is authentic.	
Demographic scales		
Nutrition knowledge (0.072)	I know quite a bit about healthy eating.	Adapted from Dodd, 2005 & Flynn et al., 1999 [41,42]
	I do not feel very knowledgeable about healthy eating.^§^	
	When it comes to healthy eating, I really don’t know a lot.^§^	
	Compared to most people, I know less about healthy eating.^§^	
Nutrition expertise (0.81)	In my circle of friends, I am one of the ‘experts’ on healthy eating.	Adapted from Dodd, 2005 & Flynn et al., 1999 [41,42]
	People seek me out for information on healthy eating.	
Healthy eating behaviour (0.92)	I intend to improve to healthiness of my diet over the next month.	Reid et al., 2015 [43]
	I plan to eat a healthier diet over the next month.	
	I want to eat a healthier diet over the next month.	
Quality of life ^‡^ (0.76)	How satisfied are you with what you are achieving in life?	Meiselman, 2016 [40]
	How satisfied are you with feeling part of your community?	
	How satisfied are you with your future security?	
	How satisfied are you with your spirituality or religion?	

SMI: social media influencer, NP: nutrition professional; ^‖^ Cronbach’s α included for both SMI and NP as separate response items were used in the questionnaire; **^¶^** Measures are based on a five-point Likert scale of strongly disagree to strongly agree; ^§^ Reversed scale items; ^‡^ Measured with ‘very dissatisfied’ to ‘very satisfied’.

**Table 3:** Descriptive statistics for scales used in the questionnaire to assess perceptions of the nutrition professional or social media influencers post; trustworthiness of post content, authenticity of post content, and perceived message appeals

	Nutrition Professional ^¶^	Social Media Influencer ^¶^	*p* ^‡^
Trustworthiness	3.8 (3.2, 4.2)	3.4 (3.0, 4.0)	0.001
Authenticity	4 (3.33, 5.0)	4 (3.0, 4.33)	0.016
Perceived message appeal			
Affiliation	4.0 (4.0, 5.0)	5.0 (4.0, 5.0)	<0.001
Hope	4.0 (4.0, 5.0)	4.0 (4.0, 5.0)	0.735
Humour	4.0 (3.0, 4.0)	4.0 (3.0, 5.0)	0.081
Heroism	4.0 (4.0, 5.0)	4.0 (3.0, 5.0)	<0.001
Ease/convenience	3.0 (2.0, 4.0)	3.0 (2.0, 4.0)	0.109
Guilt/shame	1.0 (1.0, 3.0)	1.0 (1.0, 2.0)	0.001
Sorrow	1.0 (1.0, 2.0)	1.0 (1.0, 2.0)	0.377
Fear	1.0 (1.0, 2.0)	1.0 (1.0, 2.0)	0.415

^‡^ Significance at *p* < 0.05 using the Wilcoxon signed rank test. ^¶^ Reported as median score (25th, 75th percentile) of a five-point Likert scale ranging from 1—‘strongly disagree’, to 5—‘strongly agree’.

**Table 4:** Regression results (Source × Message Appeal on Post Authenticity).

Predictors:	Beta	*t*	*p*
Dependent Variable: Post Authenticity			
Source (*X*) × Affiliation Message Appeal (*W*)	−0.12	−1.16	0.25
Source (*X*) × Hope Message Appeal (*W*)	0.15	1.46	0.15
Source (*X*) × Humour Message Appeal (*W*)	−0.04	−0.44	0.66
Source (*X*) × Heroic Message Appeal (*W*)	0.22	2.54	0.01
Source (*X*) × Convenience Message Appeal (*W*)	−0.00	−0.08	0.94
Source (*X*) × Shame Message Appeal (*W*)	0.09	0.88	0.38
Source (*X*) × Sorrow Message Appeal (*W*)	0.08	0.70	0.48
Source (*X*) × Fear Message Appeal (*W*)	−0.01	−0.04	0.96

Significance at *p* < 0.05. *X* and *W* indicate to the variables illustrated in Figure 1.

**Table 5:** Regression Results from PROCESS Model 7. Two models were developed; Model 1: post authenticity, Model 2: post trustworthiness.

Predictors:	Beta	*t*	*p*
Dependent Variable: Post Authenticity (Model 1, *R*^2^ = 0.23, *p* < 0.001)			
Source (*X*)	−0.72	−2.05	0.04
Heroic message appeal (*W*)	0.06	1.01	0.31
*X* × *W* (interaction)	0.22	2.54	0.01
Gender of participant	0.27	2.68	0.007
Source familiarity	−0.01	−0.09	0.93
Source likability	0.21	4.08	<0.001
Quality of life of participant	−0.03	−0.42	0.68
Healthy eating behaviour of participant	0.17	1.90	0.06
Subjective nutrition knowledge of participant	0.05	0.77	0.44
Subjective nutrition expertise of participant	−0.17	−3.51	<0.001
Self-reported BMI	−0.00	−0.00	0.99
Dependent Variable: Post Trustworthiness (Model 2, *R*^2^ = 0.64, *p* < 0.001)			
Source (*X*)	0.16	2.62	0.01
Post authenticity (*M*)	0.70	20.03	<0.001
Gender of participant	0.12	1.99	0.05
Source familiarity	0.05	1.59	0.11
Source likability	−0.01	−0.29	0.77
Quality of life of participant	−0.04	−1.18	0.24
Healthy eating behaviour of participant	−0.04	−0.73	0.47
Subjective nutrition knowledge of participant	0.00	0.04	0.98
Subjective nutrition expertise of participant	0.08	2.49	0.01
Self-reported BMI	−0.00	−0.14	0.89
Index of Moderated Mediation	Beta	LCI	UCI
*X* × *W*→ *M* →*Y*	0.15	0.02	0.30

Significance at *p* < 0.05. LCI = lower confidence interval. UCI = upper confidence interval. BMI: body mass index. BMI, body mass index. Letters (*M*, *W*, *X*, *Y*) are variables not abbreviations.

**Table 6:** Recommendations for nutrition professionals utilising social media platforms.

Recommendations for Nutrition Professionals Using Social Media
Use social media to communicate with young adults about health and nutrition. This study focused on Instagram posts only, which is an appropriate social media platform for young adults who mainly use Instagram and YouTube in day-to-day life.
Frame your social media messages with a heroic message appeal, focusing on bravery, nobility, and success, to increase the authenticity of your social media posts.
Be personal and share your own experiences online. Share real-life stories that are relatable to your followers to increase your perceived authenticity and trustworthiness and generate a greater persuasive capacity.

## Supplementary Materials

The following are available online at https://www.mdpi.com/2072-6643/12/6/1861/s1, (1) Questionnaire.

## Appendix A

As this is a multidisciplinary project, a glossary of terms is provided to explain terms that may be unfamiliar when used outside of either discipline.

**Term**	**Definition**
Authenticity [71]	Being true to the self in terms of an individual’s thoughts, feelings, and behaviours reflecting their true identity.
Celebrity endorsement [72]	Brands utilising a celebrity/well known persona in their advertising campaigns to materially improve financial returns.
Heroic message appeal [16]	Conveys bravery, nobility, and admiration.
Engagement [11]	Refers to social media activities that including browsing, liking, commenting and sharing content. Also referred to as passive and active social media use.
Herd behaviour [69]	The phenomenon of individuals deciding to follow others and imitating group behaviours rather than deciding independently and atomistically on the basis of their own, private information.
Instagram [73]	A social networking site that involves posting photos with the option of using enhancement filters, and nonreciprocal following of other users.
Nutrition Professionals [74]	An individual who holds a degree in Nutrition, with 3+ years experience working within the field.
Parasocial relationship [75]	A one-sided interaction where the consumer feels as if they know the celebrity well, whilst being completely unknown in return.
Post-truth era [13]	Relating to a situation in which people are more likely to accept an argument based on their emotions and beliefs, rather than one based on facts.
Self-Determination Theory [24] - Autonomy - Competence - Relatedness	A theory of human motivation. Has three components: - Autonomy: person’s need to feel that his or her activities are self-chosen, self-governed, and self-endorsed. - Competence: A person’s innate, life-span tendency to seek feelings of effectiveness, achievement, and challenge in his or her activities - Relatedness: A person’s need to feel a sense of closeness with others. This paper focuses on the perception of others being true to themselves (i.e., authentic).
Social media [18]	Any web-based communication channel, dedicated to community-based input, interaction, content-sharing or collaboration.
Social media influencer [18]	Individuals or groups of individuals who can shape attitudes and behaviours through online channels.
Source credibility [27]	A communicator’s positive characteristics that affect the receiver’s acceptance of a message. Includes the speaker’s attractiveness, trustworthiness, and expertise.
