# Supplementary material for: Strategies to Improve Health Communication: Can Health Professionals Be Heroes?
Source: Nutrients. 2020 Jun 22;12(6):1861. doi: 10.3390/nu12061861 (PMC7353280; doi:10.3390/nu12061861)
Supplement: Supplementary file 1 [file nutrients-12-01861-s001.pdf]

Authenticity & credibility survey*

**Both profiles and posts included in the survey have been anonymized. Participants saw original posts. This study focuses on the highest performing post only (first appearing post for both the social media influencer and nutrition professional on this document).*

Start of Block: Explanatory statement

Q1.1 **EXPLANATORY STATEMENT**

You are invited to take part in this survey which is part of the Communicating Health Project, a Monash University and RMIT University collaboration funded by the National Health and Medical Research Council. This survey is approved by the Monash University Human Research Ethics Committee (MUHREC) project titled ‘‘Communicating Health: perceptions of social media posts’ (project ID 19201). Please read this Explanatory Statement in full before deciding whether or not to participate in this research. If you would like further information regarding any aspect of this project, you are encouraged to contact one of the researchers: Tracy McCaffrey: tracy.mccaffrey@monash.edu, Eva Jenkins: eljen4@student.monash.edu, Jasmina Ilicic: jasmina.ilicic@monash.edu, Annika Molenaar: annika.molenaar@monash.edu We are trying to learn about how we can use social media to create and share healthy eating messages. The aim of the overall project is to understand how young adults are using social media. If you are over 18 years old, currently living in Australia and enrolled in the Monash University Department of Marketing Pilot Student Subject Pool, we would like to invite you to complete the following 20-25 minute online survey. If you decide you want to participate you will be asked your opinions on social media posts we will show you, eating, social media use, health and demographic questions including physical activity level, height and weight. The survey is anonymous and doesn’t ask you to disclose any information you could be identified with such as your name. If you decide to take part you are still free to stop at any time without giving a reason, however, your answers will not be able to be removed from the data as this survey is anonymous. If this survey brings up any issues, services available include: - Monash Nutrition Clinic for further information on your diet (has some costs involved): https://www.monash.edu/medicine/base/about/clinics/nutrition-clinic - Monash counselling services (only available to Monash staff and students): https://www.monash.edu/health/counselling - Lifeline phone line: 13 11 14 - Butterfly foundation phone line: 1800 33 4673 Data will be collected and stored on the survey software Qualtrics and stored electronically on a secure drive that only the research team has access to. Any complaints should be directed to the Executive Officer, Monash University Human Research Ethics Committee (MUHREC): Room 111, Building 3e Research Office

Monash University VIC 3800; Tel: +61 3 9905 2052; Email: muhrec@monash.edu; Fax: +61 3 9905 3831.

◯ I would like to take part and consent for my responses to be used for research purposes (1)

◯ I would not like to take part (2)

Skip To: End of Survey If EXPLANATORY STATEMENT You are invited to take part in this survey which is part of the Commu... = I would not like to take part

Q1.2 Please enter your subject ID: (4 digits)

____

End of Block: Explanatory statement

Start of Block: SMI 1 Profile

Q5.1 Please answer the following questions about this social media profile.

If you are unaware of who 'SMI'* is, then answer based on your impression of her profile in the picture.

*The social media influencers's name has been changed to 'SMI' to anonymise the person. Participants saw the real name.

Q5.2

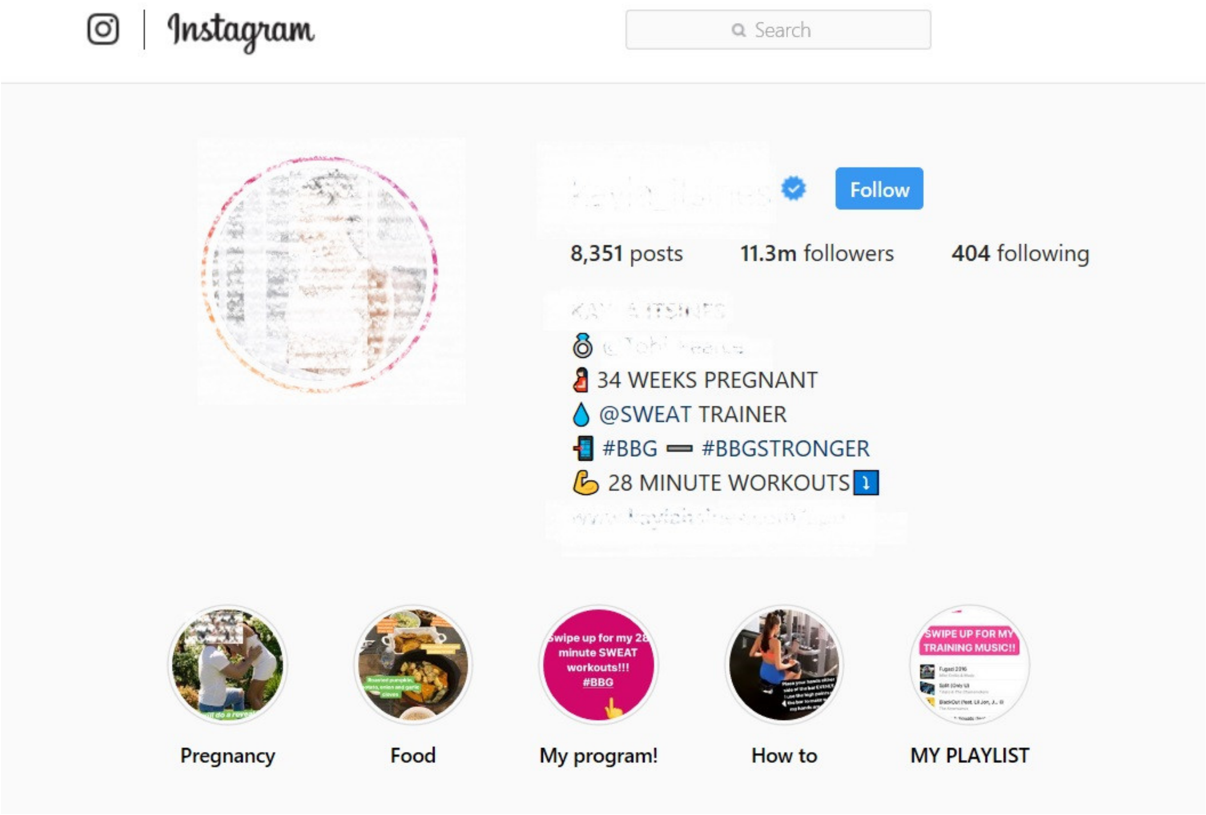
Instagram
Search
Follow
8,351 posts
11.3m followers
404 following
34 WEEKS PREGNANT
@SWEAT TRAINER
#BBG #BBGSTRONGER
28 MINUTE WORKOUTS
Pregnancy
Food
My program!
How to
MY PLAYLIST

Q5.3 Please answer the following.

	Strongly disagree (1)	2 (2)	3 (3)	4 (4)	Strongly agree (5)
I am familiar with 'SMI' (4)	○	○	○	○	○
I know a lot about 'SMI' (7)	○	○	○	○	○
I follow 'SMI' on social media (8)	○	○	○	○	○
I read her social media posts (15)	○	○	○	○	○
I find 'SMI' likeable (10)	○	○	○	○	○
My impression of 'SMI' is favourable (11)	○	○	○	○	○
I find 'SMI' warm and friendly (12)	○	○	○	○	○

Q5.4
How do you feel about 'SMI'? Please rate accordingly.

	1 (1)	2 (2)	3 (3)	4 (4)	5 (5)	
Unattractive	○	○	○	○	○	Attractive
Not classy	○	○	○	○	○	Classy
Ugly	○	○	○	○	○	Beautiful
Plain	○	○	○	○	○	Elegant
Not sexy	○	○	○	○	○	Sexy

Q5.5
How do you feel about 'SMI' in regard to her understanding of nutrition? Please rate accordingly.

	1 (1)	2 (2)	3 (3)	4 (4)	5 (5)	
Not an expert	○	○	○	○	○	Expert
Inexperienced	○	○	○	○	○	Experienced
Unknowledgeable	○	○	○	○	○	Knowledgeable
Unqualified	○	○	○	○	○	Qualified
Unskilled	○	○	○	○	○	Skilled

Q5.6 How much do you agree with the following statements?

	Strongly disagree (6)	2 (7)	3 (8)	4 (9)	Strongly agree (10)
I find 'SMI' trustworthy (1)	○	○	○	○	○
She would be honest in her recommendations (2)	○	○	○	○	○
She appears to be dependable (3)	○	○	○	○	○
She seems to be sincere (4)	○	○	○	○	○
She appears to be reliable (5)	○	○	○	○	○

Q5.7 How much do you agree with the following statements?

	Strongly disagree (1)	2 (2)	3 (3)	4 (4)	Strongly agree (5)
'SMI' tries to act in a manner that is consistent with her held values, even if others criticise or reject her for doing so (1)	○	○	○	○	○
'SMI' cares about openness and honesty in close relationships with others (2)	○	○	○	○	○
In general, 'SMI' places a good deal of importance on others understanding who she truly is (3)	○	○	○	○	○
People can count on 'SMI' being who she is regardless of the situation (4)	○	○	○	○	○

Q5.8 How much do you agree with the following statements?

	Strongly disagree (1)	2 (2)	3 (3)	4 (4)	Strongly agree (5)
'SMI' seems like an expert in healthy eating/diet/fitness (1)	○	○	○	○	○
She is experienced in the topic of healthy eating/diet/fitness (2)	○	○	○	○	○
'SMI' is qualified to talk about and give advice on health/food (3)	○	○	○	○	○
She is skilled in this area of health/food/fitness (4)	○	○	○	○	○
When it comes to health/food topics, 'SMI' is knowledgeable (5)	○	○	○	○	○

Page Break

Page Break

End of Block: SMI 1 Profile

Start of Block: SMI 1 Post 1

Q181 Please answer the following questions about this social media post.

If you are unaware of who 'SMI' is, then answer based on your impression from the post.

Q6.1

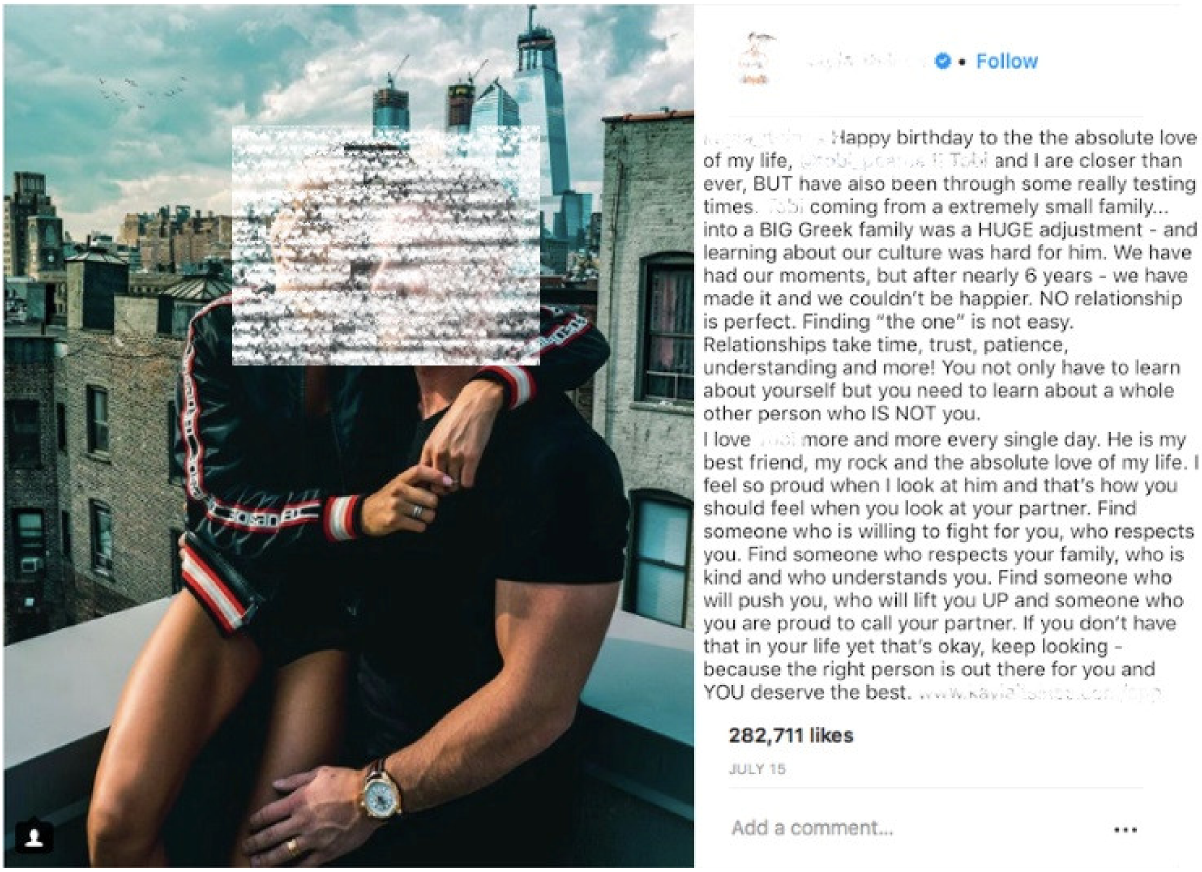

Q6.2 Which emotions do you feel after seeing/reading this post?

	Strongly disagree (1)	2 (2)	3 (3)	4 (4)	Strongly agree (5)
**Affiliation**: Feelings of love, belonging and togetherness (1)	○	○	○	○	○
**Hope:** Feelings of hope and reassurance (2)	○	○	○	○	○
**Humour:** Feelings of fun, happiness, laughter, joy, light-heartedness, cheerfulness, comedy (3)	○	○	○	○	○
**Heroic/successful:** Feelings of bravery, nobility, admiration (4)	○	○	○	○	○
**Ease/convenience:** Feeling that little effort is required to engage in the behaviour in the post (6)	○	○	○	○	○
**Guilt/shame:** Feelings of shame and remorse (7)	○	○	○	○	○
**Sorrow:** Feelings of sadness, worry, loss, misery and unhappiness (8)	○	○	○	○	○
**Fear:** Feelings of terror, dread, anxiety, panic and alarm (9)	○	○	○	○	○

Q6.3 Looking/reading this social media post, what do you think 'SMI' is trying to achieve? How much do you agree with the following statements?

	Strongly disagree (1)	2 (2)	3 (3)	4 (4)	Strongly agree (5)
'SMI' is trying to sell me something (1)	○	○	○	○	○
'SMI' is encouraging me to do something, such as changing my behaviour for the better (2)	○	○	○	○	○
'SMI' is sharing her opinion and feelings about something (3)	○	○	○	○	○
'SMI' is providing me with useful information and facts about health/food (4)	○	○	○	○	○

Q6.4 How much do you agree with the following statements?

	Strongly disagree (1)	2 (2)	3 (3)	4 (4)	Strongly agree (5)
The image used for this post is attractive (1)	o	o	o	o	o
I find this post classy (2)	o	o	o	o	o
This post is plain (3)	o	o	o	o	o
The image used for this post is sexy (4)	o	o	o	o	o
I find the image in this post beautiful (5)	o	o	o	o	o

Q6.5 How much do you agree with the following statements?

	Strongly disagree (1)	2 (2)	3 (3)	4 (4)	Strongly agree (5)
The content of this post seems dependable (1)	○	○	○	○	○
I find the information provided in this post reliable (2)	○	○	○	○	○
This post is being honest in its recommendations (3)	○	○	○	○	○
This post seems sincere (4)	○	○	○	○	○
I trust the information provided in this post (5)	○	○	○	○	○

Q6.6 How much do you agree with the following statements?

	Strongly disagree (1)	2 (2)	3 (3)	4 (4)	Strongly agree (5)
The content in this post is genuine (1)	○	○	○	○	○
The content in this post seems real to me (2)	○	○	○	○	○
This content in this post is authentic (3)	○	○	○	○	○

Q6.7 What emotions do you think the post is trying to show?

	Strongly disagree (1)	2 (2)	3 (3)	4 (4)	Strongly agree (5)
**Affiliation**: Feelings of love, belonging and togetherness (1)	○	○	○	○	○
**Hope:** Feelings of hope and reassurance (2)	○	○	○	○	○
**Humour:** Feelings of fun, happiness, laughter, joy, light-heartedness, cheerfulness, comedy (3)	○	○	○	○	○
**Heroism/success:** Feelings of bravery, nobility, admiration (4)	○	○	○	○	○
**Ease/convenience:** Feeling that little effort is required to engage in the behaviour in the post (6)	○	○	○	○	○
**Guilt/shame:** Feelings of shame and remorse (7)	○	○	○	○	○
**Sorrow:** Feelings of sadness, worry, loss, misery and unhappiness (8)	○	○	○	○	○
**Fear:** Feelings of terror, dread, anxiety, panic and alarm (9)	○	○	○	○	○

End of Block: SMI 1 Post 1

Start of Block: SMI 1 Post 2

Q182 Please answer the following questions about this social media post.

If you are unaware of who 'SMI' is, then answer based on your impression from the post.

Q7.1

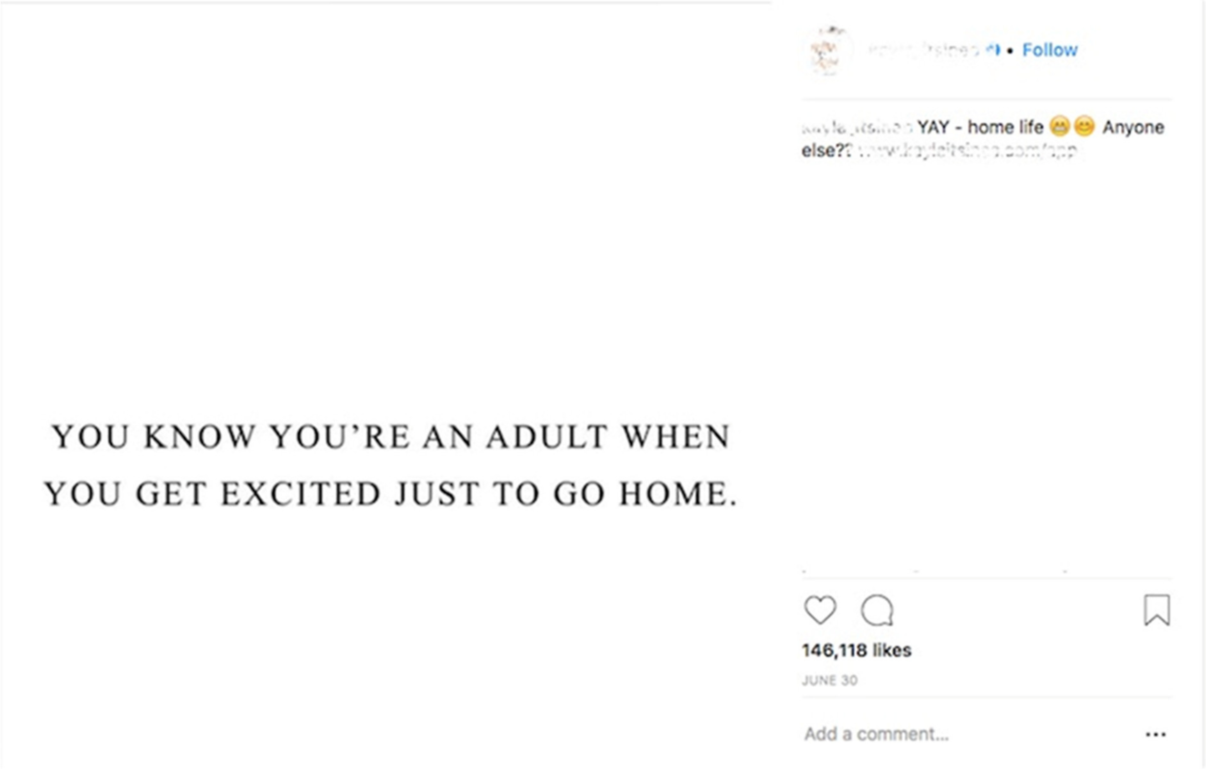

Q7.2 Which emotions do you feel after seeing/reading this post?

	Strongly disagree (1)	2 (2)	3 (3)	4 (4)	Strongly agree (5)
**Affiliation**: Feelings of love, belonging and togetherness (1)	○	○	○	○	○
**Hope:** Feelings of hope and reassurance (2)	○	○	○	○	○
**Humour:** Feelings of fun, happiness, laughter, joy, light-heartedness, cheerfulness, comedy (3)	○	○	○	○	○
**Heroic/successful:** Feelings of bravery, nobility, admiration (4)	○	○	○	○	○
**Ease/convenience:** Feeling that little effort is required to engage in the behaviour in the post (6)	○	○	○	○	○
**Guilt/shame:** Feelings of shame and remorse (7)	○	○	○	○	○
**Sorrow:** Feelings of sadness, worry, loss, misery and unhappiness (8)	○	○	○	○	○
**Fear:** Feelings of terror, dread, anxiety, panic and alarm (9)	○	○	○	○	○

Q7.3 Looking/reading this social media post, what do you think 'SMI’ is trying to achieve? How much do you agree with the following statements?

	Strongly disagree (1)	2 (2)	3 (3)	4 (4)	Strongly agree (5)
'SMI’ is trying to sell me something (1)	○	○	○	○	○
'SMI’ is encouraging me to do something, such as changing my behaviour for the better (2)	○	○	○	○	○
'SMI’ is sharing her opinion and feelings about something (3)	○	○	○	○	○
'SMI’ is providing me with useful information and facts about health/food (4)	○	○	○	○	○

Q7.4 How much do you agree with the following statements?

	Strongly disagree (1)	2 (2)	3 (3)	4 (4)	Strongly agree (5)
The image used for this post is attractive (1)	○	○	○	○	○
I find this post classy (2)	○	○	○	○	○
This post is plain (3)	○	○	○	○	○
The image used for this post is sexy (4)	○	○	○	○	○
I find the image in this post beautiful (5)	○	○	○	○	○

Q7.5 How much do you agree with the following statements?

	Strongly disagree (1)	2 (2)	3 (3)	4 (4)	Strongly agree (5)
The content of this post seems dependable (1)	o	o	o	o	o
I find the information provided in this post reliable (2)	o	o	o	o	o
This post is being honest in its recommendations (3)	o	o	o	o	o
This post seems sincere (4)	o	o	o	o	o
I trust the information provided in this post (5)	o	o	o	o	o

Q7.6 How much do you agree with the following statements?

	Strongly disagree (1)	2 (2)	3 (3)	4 (4)	Strongly agree (5)
The content in this post is genuine (1)	o	o	o	o	o
The content in this post seems real to me (2)	o	o	o	o	o
This content in this post is authentic (3)	o	o	o	o	o

Q7.7 What emotions do you think the post is trying to show?

	Strongly disagree (1)	2 (2)	3 (3)	4 (4)	Strongly agree (5)
**Affiliation**: Feelings of love, belonging and togetherness (1)	○	○	○	○	○
**Hope:** Feelings of hope and reassurance (2)	○	○	○	○	○
**Humour:** Feelings of fun, happiness, laughter, joy, light-heartedness, cheerfulness, comedy (3)	○	○	○	○	○
**Heroism/success:** Feelings of bravery, nobility, admiration (4)	○	○	○	○	○
**Ease/convenience:** Feeling that little effort is required to engage in the behaviour in the post (6)	○	○	○	○	○
**Guilt/shame:** Feelings of shame and remorse (7)	○	○	○	○	○
**Sorrow:** Feelings of sadness, worry, loss, misery and unhappiness (8)	○	○	○	○	○
**Fear:** Feelings of terror, dread, anxiety, panic and alarm (9)	○	○	○	○	○

End of Block: SMI 1 Post 2

Start of Block: NutrProf 1 Profile

Q2.1 Please answer the following questions about this social media profile.

If you are unaware of who 'NP' is, then answer based on your impression of her profile in the picture.
*NP has been used to anonymize the profile for the purposes of this document. Participants saw the original version.

Q2.2

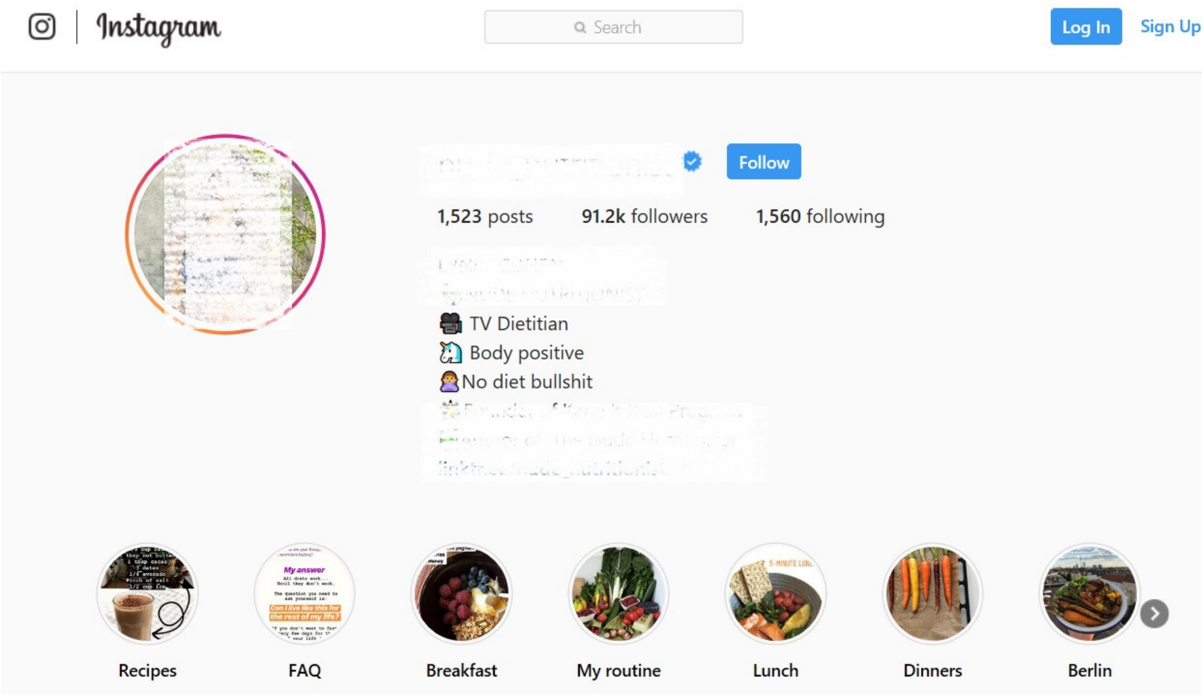

Q2.3 Please answer the following.

	Strongly disagree (1)	2 (2)	3 (3)	4 (4)	Strongly agree (5)
I am familiar with 'NP' (4)	○	○	○	○	○
I know a lot about 'NP' (7)	○	○	○	○	○
I follow 'NP' on social media (8)	○	○	○	○	○
I read her social media posts (16)	○	○	○	○	○
I find 'NP' likeable (10)	○	○	○	○	○
My impression of 'NP' is favourable (11)	○	○	○	○	○
I find 'NP' warm and friendly (12)	○	○	○	○	○

Q2.4 How do you feel about 'NP'? Please rate accordingly.

	1 (1)	2 (2)	3 (3)	4 (4)	5 (5)	
Unattractive	○	○	○	○	○	Attractive
Not classy	○	○	○	○	○	Classy
Ugly	○	○	○	○	○	Beautiful
Plain	○	○	○	○	○	Elegant
Not sexy	○	○	○	○	○	Sexy

Q2.5
How do you feel about ‘NP’ in regard to her understanding of nutrition? Please rate accordingly.

	1 (1)	2 (2)	3 (3)	4 (4)	5 (5)	
Not an expert	○	○	○	○	○	Expert
Inexperienced	○	○	○	○	○	Experienced
Unknowledgeable	○	○	○	○	○	Knowledgeable
Unqualified	○	○	○	○	○	Qualified
Unskilled	○	○	○	○	○	Skilled

Q2.6 How much do you agree with the following statements?

	Strongly disagree (1)	2 (2)	3 (3)	4 (4)	Strongly agree (5)
I find ‘NP’ trustworthy (1)	○	○	○	○	○
She would be honest in her recommendations (2)	○	○	○	○	○
She appears to be dependable (3)	○	○	○	○	○
She seems to be sincere (4)	○	○	○	○	○
She appears to be reliable (5)	○	○	○	○	○

Q2.7 How much do you agree with the following statements?

	Strongly disagree (1)	2 (2)	3 (3)	4 (4)	Strongly agree (5)
'NP' tries to act in a manner that is consistent with her held values, even if others criticise or reject her for doing so (1)	◯	◯	◯	◯	◯
'NP' cares about openness and honesty in close relationships with others (2)	◯	◯	◯	◯	◯
In general, 'NP' places a good deal of importance on others understanding who she truly is (3)	◯	◯	◯	◯	◯
People can count on 'NP' being who she is regardless of the situation (4)	◯	◯	◯	◯	◯

Q2.8 How much do you agree with the following statements?

	Strongly disagree (1)	2 (2)	3 (3)	4 (4)	Strongly agree (5)
‘NP’ seems like an expert in healthy eating/diet/fitness (1)	○	○	○	○	○
She is experienced in the topic of healthy eating/diet/fitness (2)	○	○	○	○	○
‘NP’ is qualified to talk about and give advice on health/food (3)	○	○	○	○	○
She is skilled in this area of health/food/fitness (4)	○	○	○	○	○
When it comes to health/food topics, ‘NP’ is knowledgeable (5)	○	○	○	○	○

Page Break

Page Break

End of Block: NutrProf 1 Profile

Start of Block: NutrProf 1 Post 1

Q183 Please answer the following questions about this social media post.

If you are unaware of who 'NP' is, then answer based on your impression from the post.

Q3.1

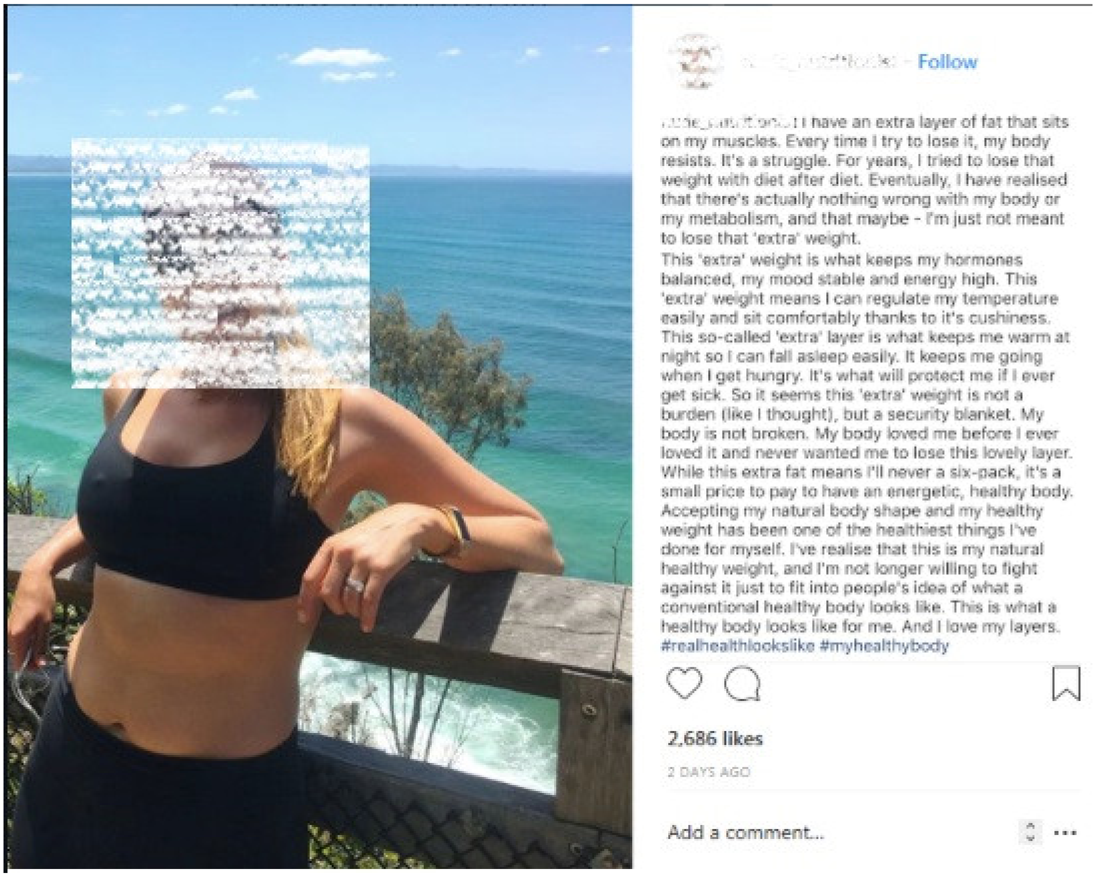

Q3.2 Which emotions do you feel after seeing/reading this post?

	Strongly disagree (1)	2 (2)	3 (3)	4 (4)	Strongly agree (5)
**Affiliation**: Feelings of love, belonging and togetherness (1)	○	○	○	○	○
**Hope:** Feelings of hope and reassurance (2)	○	○	○	○	○
**Humour:** Feelings of fun, happiness, laughter, joy, light-heartedness, cheerfulness, comedy (3)	○	○	○	○	○
**Heroic/successful:** Feelings of bravery, nobility, admiration (4)	○	○	○	○	○
**Ease/convenience:** Feeling that little effort is required to engage in the behaviour in the post (6)	○	○	○	○	○
**Guilt/shame:** Feelings of shame and remorse (7)	○	○	○	○	○
**Sorrow:** Feelings of sadness, worry, loss, misery and unhappiness (8)	○	○	○	○	○
**Fear:** Feelings of terror, dread, anxiety, panic and alarm (9)	○	○	○	○	○

Q3.3 Looking at/reading this social media post, what do you think 'NP' is trying to achieve? How much do you agree with the following statements?

	Strongly disagree (1)	2 (2)	3 (3)	4 (4)	Strongly agree (5)
‘NP’ is trying to sell me something (1)	○	○	○	○	○
‘NP’ is encouraging me to do something, such as changing my behaviour for the better (2)	○	○	○	○	○
‘NP’ is sharing her opinion and feelings about something (3)	○	○	○	○	○
‘NP’ is providing me with useful information and facts about health/food (4)	○	○	○	○	○

Q3.4 How much do you agree with the following statements?

	Strongly disagree (1)	2 (2)	3 (3)	4 (4)	Strongly agree (5)
The image used for this post is attractive (1)	o	o	o	o	o
I find this post classy (2)	o	o	o	o	o
This post is plain (3)	o	o	o	o	o
The image used for this post is sexy (4)	o	o	o	o	o
I find the image in this post beautiful (5)	o	o	o	o	o

Q3.5 How much do you agree with the following statements?

	Strongly disagree (1)	2 (2)	3 (3)	4 (4)	Strongly agree (5)
The content of this post seems dependable (1)	○	○	○	○	○
I find the information provided in this post reliable (2)	○	○	○	○	○
This post is being honest in its recommendations (3)	○	○	○	○	○
This post seems sincere (4)	○	○	○	○	○
I trust the information provided in this post (5)	○	○	○	○	○

Q3.6 How much do you agree with the following statements?

	Strongly disagree (1)	2 (2)	3 (3)	4 (4)	Strongly agree (5)
The content in this post is genuine (1)	○	○	○	○	○
The content in this post seems real to me (2)	○	○	○	○	○
This content in this post is authentic (3)	○	○	○	○	○

Q3.7 What emotions do you think the post is trying to show?

	Strongly disagree (1)	2 (2)	3 (3)	4 (4)	Strongly agree (5)
**Affiliation**: Feelings of love, belonging and togetherness (1)	○	○	○	○	○
**Hope:** Feelings of hope and reassurance (2)	○	○	○	○	○
**Humour:** Feelings of fun, happiness, laughter, joy, light-heartedness, cheerfulness, comedy (3)	○	○	○	○	○
**Heroism/success:** Feelings of bravery, nobility, admiration (4)	○	○	○	○	○
**Ease/convenience:** Feeling that little effort is required to engage in the behaviour in the post (6)	○	○	○	○	○
**Guilt/shame:** Feelings of shame and remorse (7)	○	○	○	○	○
**Sorrow:** Feelings of sadness, worry, loss, misery and unhappiness (8)	○	○	○	○	○
**Fear:** Feelings of terror, dread, anxiety, panic and alarm (9)	○	○	○	○	○

End of Block: NutrProf 1 Post 1

Start of Block: NutrProf 1 Post 2

Q184 Please answer the following questions about this social media post.

If you are unaware of who ‘NP’ is, then answer based on your impression from the post.

Q4.1

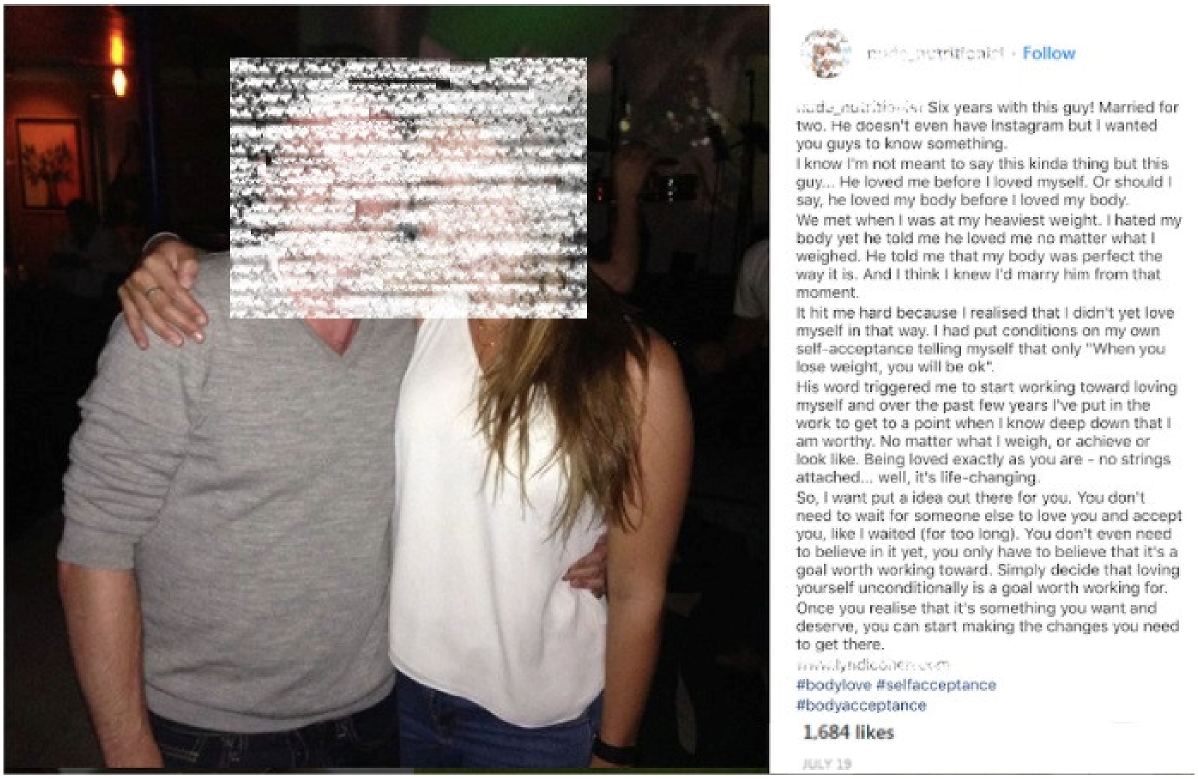

Q4.2 Which emotions do you feel after seeing/reading this post?

	Strongly disagree (1)	2 (2)	3 (3)	4 (4)	Strongly agree (5)
**Affiliation**: Feelings of love, belonging and togetherness (1)	○	○	○	○	○
**Hope:** Feelings of hope and reassurance (2)	○	○	○	○	○
**Humour:** Feelings of fun, happiness, laughter, joy, light-heartedness, cheerfulness, comedy (3)	○	○	○	○	○
**Heroic/successful:** Feelings of bravery, nobility, admiration (4)	○	○	○	○	○
**Ease/convenience:** Feeling that little effort is required to engage in the behaviour in the post (6)	○	○	○	○	○
**Guilt/shame:** Feelings of shame and remorse (7)	○	○	○	○	○
**Sorrow:** Feelings of sadness, worry, loss, misery and unhappiness (8)	○	○	○	○	○
**Fear:** Feelings of terror, dread, anxiety, panic and alarm (9)	○	○	○	○	○

Q4.3 Looking/reading this social media post, what do you think 'NP' is trying to achieve? How much do you agree with the following statements?

	Strongly disagree (1)	2 (2)	3 (3)	4 (4)	Strongly agree (5)
'NP' is trying to sell me something (1)	o	o	o	o	o
'NP' is encouraging me to do something, such as changing my behaviour for the better (2)	o	o	o	o	o
'NP' is sharing her opinion and feelings about something (3)	o	o	o	o	o
'NP' is providing me with useful information and facts about health/food (4)	o	o	o	o	o

Q4.4 How much do you agree with the following statements?

	Strongly disagree (11)	2 (13)	3 (14)	4 (18)	Strongly agree (19)
The content of this post seems dependable (1)	○	○	○	○	○
I find the information provided in this post reliable (2)	○	○	○	○	○
This post is being honest in its recommendations (3)	○	○	○	○	○
This post seems sincere (4)	○	○	○	○	○
I trust the information provided in this post (5)	○	○	○	○	○

Q4.5 How much do you agree with the following statements?

	Strongly disagree (1)	2 (2)	3 (3)	4 (4)	Strongly agree (5)
The image used for this post is attractive (1)	○	○	○	○	○
I find this post classy (2)	○	○	○	○	○
This post is plain (3)	○	○	○	○	○
The image used for this post is sexy (4)	○	○	○	○	○
I find the image in this post beautiful (5)	○	○	○	○	○

Q4.6 How much do you agree with the following statements?

	Strongly disagree (1)	2 (2)	3 (3)	4 (4)	Strongly agree (5)
The content in this post is genuine (1)	○	○	○	○	○
The content in this post seems real to me (2)	○	○	○	○	○
The content in this post is authentic (3)	○	○	○	○	○

Q4.7 What emotions do you think the post is trying to show?

	Strongly disagree (1)	2 (2)	3 (3)	4 (4)	Strongly agree (5)
**Affiliation**: Feelings of love, belonging and togetherness (1)	○	○	○	○	○
**Hope:** Feelings of hope and reassurance (2)	○	○	○	○	○
**Humour:** Feelings of fun, happiness, laughter, joy, light-heartedness, cheerfulness, comedy (3)	○	○	○	○	○
**Heroism/success:** Feelings of bravery, nobility, admiration (4)	○	○	○	○	○
**Ease/convenience:** Feeling that little effort is required to engage in the behaviour in the post (6)	○	○	○	○	○
**Guilt/shame:** Feelings of shame and remorse (7)	○	○	○	○	○
**Sorrow:** Feelings of sadness, worry, loss, misery and unhappiness (8)	○	○	○	○	○
**Fear:** Feelings of terror, dread, anxiety, panic and alarm (9)	○	○	○	○	○

End of Block: NutrProf 1 Post 2

Start of Block: 1. Demographics

Q8.1 Please tell us a little bit about yourself.

Q8.2 What is your age?

- ◯ 18 (46)
- ◯ 19 (65)
- ◯ 20 (66)
- ◯ 21 (67)
- ◯ 22 (68)
- ◯ 23 (69)
- ◯ 24 (70)
- ◯ 25 (71)
- ◯ 26 (72)
- ◯ 27 (73)
- ◯ 28 (74)
- ◯ 29 (75)
- ◯ 30 (76)
- ◯ 31 (77)
- ◯ 32 (78)
- ◯ 33 (79)
- ◯ 34 (80)
- ◯ 35 (81)
- ◯ 36 (82)
- ◯ 37 (83)
- ◯ 38 (84)

- ○ 39 (85)
- ○ 40 (86)
- ○ 41 (87)
- ○ 42 (88)
- ○ 43 (89)
- ○ 44 (90)
- ○ 45 (91)
- ○ 46 (92)
- ○ 47 (93)
- ○ 48 (94)
- ○ 49 (95)
- ○ 50 (96)
- ○ 51 (97)
- ○ 52 (98)
- ○ 53 (99)
- ○ 54 (100)
- ○ 55 (101)
- ○ 56 (102)
- ○ 57 (103)
- ○ 58 (104)
- ○ 59 (105)

○ 60 (106)

○ 61 (107)

○ 62 (108)

○ 63 (109)

○ 64 (110)

○ >65 (111)

Q8.3 I identify as...

○ Male (1)

○ Female (2)

○ Trans male/trans man (3)

○ Trans female/trans woman (4)

○ Non-binary/gender fluid (5)

○ My gender is not listed (6) ____

○ Prefer not to say (7)

Q8.4 What country were you born in?

○ Australia (1)

○ Other (2)

Display This Question:

If What country were you born in? = Other

X→

Q8.5 Other, please specify

- ◯ Afghanistan (1)
- ◯ Albania (2)
- ◯ Algeria (3)
- ◯ Andorra (4)
- ◯ Angola (5)
- ◯ Antigua and Barbuda (6)
- ◯ Argentina (7)
- ◯ Armenia (8)
- ◯ Australia (9)
- ◯ Austria (10)
- ◯ Azerbaijan (11)
- ◯ Bahamas (12)
- ◯ Bahrain (13)
- ◯ Bangladesh (14)
- ◯ Barbados (15)
- ◯ Belarus (16)
- ◯ Belgium (17)
- ◯ Belize (18)
- ◯ Benin (19)
- ◯ Bhutan (20)
- ◯ Bolivia (21)

- Bosnia and Herzegovina (22)
- Botswana (23)
- Brazil (24)
- Brunei Darussalam (25)
- Bulgaria (26)
- Burkina Faso (27)
- Burundi (28)
- Cambodia (29)
- Cameroon (30)
- Canada (31)
- Cape Verde (32)
- Central African Republic (33)
- Chad (34)
- Chile (35)
- China (36)
- Colombia (37)
- Comoros (38)
- Congo, Republic of the... (39)
- Costa Rica (40)
- Côte d'Ivoire (41)
- Croatia (42)

- Cuba (43)
- Cyprus (44)
- Czech Republic (45)
- Democratic People's Republic of Korea (46)
- Democratic Republic of the Congo (47)
- Denmark (48)
- Djibouti (49)
- Dominica (50)
- Dominican Republic (51)
- Ecuador (52)
- Egypt (53)
- El Salvador (54)
- Equatorial Guinea (55)
- Eritrea (56)
- Estonia (57)
- Ethiopia (58)
- Fiji (59)
- Finland (60)
- France (61)
- Gabon (62)
- Gambia (63)

- Georgia (64)
- Germany (65)
- Ghana (66)
- Greece (67)
- Grenada (68)
- Guatemala (69)
- Guinea (70)
- Guinea-Bissau (71)
- Guyana (72)
- Haiti (73)
- Honduras (74)
- Hong Kong (S.A.R.) (75)
- Hungary (76)
- Iceland (77)
- India (78)
- Indonesia (79)
- Iran, Islamic Republic of... (80)
- Iraq (81)
- Ireland (82)
- Israel (83)
- Italy (84)

- Jamaica (85)
- Japan (86)
- Jordan (87)
- Kazakhstan (88)
- Kenya (89)
- Kiribati (90)
- Kuwait (91)
- Kyrgyzstan (92)
- Lao People's Democratic Republic (93)
- Latvia (94)
- Lebanon (95)
- Lesotho (96)
- Liberia (97)
- Libyan Arab Jamahiriya (98)
- Liechtenstein (99)
- Lithuania (100)
- Luxembourg (101)
- Madagascar (102)
- Malawi (103)
- Malaysia (104)
- Maldives (105)

- Mali (106)
- Malta (107)
- Marshall Islands (108)
- Mauritania (109)
- Mauritius (110)
- Mexico (111)
- Micronesia, Federated States of... (112)
- Monaco (113)
- Mongolia (114)
- Montenegro (115)
- Morocco (116)
- Mozambique (117)
- Myanmar (118)
- Namibia (119)
- Nauru (120)
- Nepal (121)
- Netherlands (122)
- New Zealand (123)
- Nicaragua (124)
- Niger (125)
- Nigeria (126)

- North Korea (127)
- Norway (128)
- Oman (129)
- Pakistan (130)
- Palau (131)
- Panama (132)
- Papua New Guinea (133)
- Paraguay (134)
- Peru (135)
- Philippines (136)
- Poland (137)
- Portugal (138)
- Qatar (139)
- Republic of Korea (140)
- Republic of Moldova (141)
- Romania (142)
- Russian Federation (143)
- Rwanda (144)
- Saint Kitts and Nevis (145)
- Saint Lucia (146)
- Saint Vincent and the Grenadines (147)

- Samoa (148)
- San Marino (149)
- Sao Tome and Principe (150)
- Saudi Arabia (151)
- Senegal (152)
- Serbia (153)
- Seychelles (154)
- Sierra Leone (155)
- Singapore (156)
- Slovakia (157)
- Slovenia (158)
- Solomon Islands (159)
- Somalia (160)
- South Africa (161)
- South Korea (162)
- Spain (163)
- Sri Lanka (164)
- Sudan (165)
- Suriname (166)
- Swaziland (167)
- Sweden (168)

- Switzerland (169)
- Syrian Arab Republic (170)
- Tajikistan (171)
- Thailand (172)
- The former Yugoslav Republic of Macedonia (173)
- Timor-Leste (174)
- Togo (175)
- Tonga (176)
- Trinidad and Tobago (177)
- Tunisia (178)
- Turkey (179)
- Turkmenistan (180)
- Tuvalu (181)
- Uganda (182)
- Ukraine (183)
- United Arab Emirates (184)
- United Kingdom of Great Britain and Northern Ireland (185)
- United Republic of Tanzania (186)
- United States of America (187)
- Uruguay (188)
- Uzbekistan (189)

○ Vanuatu (190)

○ Venezuela, Bolivarian Republic of... (191)

○ Viet Nam (192)

○ Yemen (193)

○ Zambia (580)

○ Zimbabwe (1357)

Display This Question:

If What country were you born in? = Other

JS

Q8.6 What year did you arrive in Australia?

Year (3)	

(Table Truncated to 63 Columns)

Page Break

Q8.7 I am currently...

- Working full-time (1)
- Working part-time (2)
- Working casually (3)
- Not working (4)
- Prefer not to say (5)

Q8.8 I am currently...

- Studying full-time (1)
- Studying part-time (2)
- Prefer not to say (4)

Display This Question:

If I am currently... = Working full-time

Or I am currently... = Working part-time

Or I am currently... = Working casually

Q8.9 What is your occupation?

○ Professional eg. Teacher, Nurse, Accountant, Engineer, Journalist (1)

○ Clerical or Administrative Worker eg. Secretary, Numerical clerk, Personal assistant (2)

○ Technician or Trades Worker eg. Carpenter, Plumber, Mechanic, Science technician (3)

○ Manager eg. General manager, Construction manager, Retail manager, Farm manager (4)

○ Community or Personal Service Worker eg. Hospitality worker, Carer, Personal trainer, Health care support (5)

○ Labourer eg. Cleaner, Food preparation assistant, Factory process worker, Building or plumbing labourer (6)

○ Sales Worker eg. Sales representative, Sales assistant (7)

○ Machinery Operator or Driver eg. Machine operator, Road or rail driver (8)

○ Prefer not to say (10)

Q8.10 What is your **weekly** gross income (**before** tax, superannuation, salary sacrifice and any other automatic deductions)?

- ◯ No income (1)
- ◯ $1 - $149 weekly ($1-$7,799 per year) (2)
- ◯ $150 - $299 weekly ($7,800-$15,599 per year) (3)
- ◯ $300 - $399 weekly ($15,600-$20,799 per year) (4)
- ◯ $400 - $499 weekly ($20,800-$25,999 per year) (5)
- ◯ $500 - $649 weekly ($26,000-$33,799 per year) (6)
- ◯ $650 - $799 weekly ($33,800-$41,599 per year) (7)
- ◯ $800 - $999 weekly ($41,600-$51,999 per year) (8)
- ◯ $1,000 - $1,249 weekly ($52,000-$64,999 per year) (9)
- ◯ $1,250 - $1,499 weekly ($65,000-$77,999 per year) (10)
- ◯ $1,500 - $1,749 weekly ($78,000-$90,999 per year) (11)
- ◯ $1,750 - $1,999 weekly ($91,000-$103,999 per year) (12)
- ◯ $2,000 - $2,999 weekly ($104,000-$155,999 per year) (13)
- ◯ $3,000 or more weekly ($156,000 or more per year) (14)
- ◯ Prefer not to say (15)

Q8.11 Who do you live with currently? (Please tick all that apply)

- ☐ Alone (1)
- ☐ Parent(s) (2)
- ☐ My partner (3)
- ☐ Friend(s)/housemate(s) (4)
- ☐ My child(ren) (5)
- ☐ Other family (6)
- ☐ I don't wish to say (7)

Page Break

Q8.12 As this survey is about healthy eating lifestyles, we would like to know your weight and height as a way to estimate body mass index (BMI). Please be assured that your responses will remain anonymous and confidential.

Q8.13 Your weight in kilograms (kg)

____

Q8.14 Your height in centimetres (cm)

____

End of Block: 1. Demographics

Start of Block: 2. Quality of life and physical activity

Q9.1
Please tell us more about your health related values and physical activity level.

Q9.2 Please rate the following questions based on your satisfaction.

	Very poor / very dissatisfied (1)	2 (2)	3 (3)	4 (4)	Very good / very satisfied (5)
How satisfied are you with what you are achieving in life? (13)	○	○	○	○	○
How satisfied are you with feeling part of your community? (6)	○	○	○	○	○
How satisfied are you with your future security? (9)	○	○	○	○	○
How satisfied are you with your spirituality or religion? (10)	○	○	○	○	○

Q9.3 Would you say that you are physically more active, less active or about as active as other persons your age?

○ More (1)

○ Less (2)

○ Same (3)

End of Block: 2. Quality of life and physical activity

Start of Block: 3. Healthy eating and consumption

Q10.1 Please tell us more about your diet and health-related beliefs.

Q10.2 Which of these statements do you identify with the most? (Choose **one**)

○ **I'm passionate about healthy eating and health plays a big part in my life**. I use social media to follow active lifestyle personalities or get new recipes/exercise ideas. I may even buy superfoods or follow a particular type of diet. I like to think I am super healthy. (1)

○ **I'm health-conscious and being healthy and eating healthy is important to me.** Although health means different things to different people, I make conscious lifestyle decisions about eating based on what I believe healthy means. I look for new recipes and healthy eating information on social media. (2)

○ **I aspire to be healthy (but struggle sometimes).** Healthy eating is hard work! I've tried to improve my diet, but always find things that make it difficult to stick with the changes. Sometimes I notice recipe ideas or healthy eating hacks, and if it seems easy enough, I'll give it a go. (3)

○ **I try and live a balanced lifestyle, and I think that all foods are okay in moderation.** I shouldn't have to feel guilty about eating a piece of cake now and again. I get all sorts of inspiration from social media like finding out about new restaurants, fun recipes and sometimes healthy eating tips. (4)

○ **I'm contemplating healthy eating but it's not a priority for me right now.** I know the basics about what it means to be healthy, but it doesn't seem relevant to me right now. I have taken a few steps to be healthier but I am not motivated to make it a high priority because I have too many other things going on in my life. (5)

○ **I'm not bothered about healthy eating.** I don't really see the point and I don't think about it. I don't really notice healthy eating tips or recipes and I don't care what I eat. (6)

Page Break

Q10.3 How much do you agree/disagree with the following statements about healthy eating?

	Strongly disagree (1)	2 (2)	3 (3)	4 (4)	Strongly agree (5)
I know quite a bit about healthy eating (1)	○	○	○	○	○
I do not feel very knowledgeable about healthy eating (2)	○	○	○	○	○
When it comes to healthy eating, I really don't know a lot (3)	○	○	○	○	○
In my circle of friends, I am one of the "experts" on healthy eating (4)	○	○	○	○	○
Compared to most people, I know less about healthy eating (5)	○	○	○	○	○
People seek me out for information on healthy eating (6)	○	○	○	○	○

Q11.1 How much do you agree/disagree with the following statements about the healthiness of your diet?

	Strongly disagree (1)	2 (2)	3 (3)	4 (4)	Strongly agree (5)
I currently eat healthily (1)	○	○	○	○	○
I am satisfied with the healthiness of my diet (2)	○	○	○	○	○
I don't need to improve the healthiness of my diet (6)	○	○	○	○	○
I intend to improve the healthiness of my diet over the next month (3)	○	○	○	○	○
I plan to eat a healthier diet over the next month (4)	○	○	○	○	○
I want to eat a healthier diet over the next month (5)	○	○	○	○	○

Page Break

End of Block: 3. Healthy eating and consumption

Start of Block: 5. Online and social media behaviour

Q12.1 Please tell us more about your social media usage and online behaviour.

Q12.2 How much do you agree with the following statements about social media?

	Strongly disagree (1)	2 (2)	3 (3)	4 (4)	Strongly agree (5)
Social media helps me to be the type of person I want to be (6)	○	○	○	○	○
My social media profile is the real 'me' (7)	○	○	○	○	○
My social media profile is what I want people to see about me (8)	○	○	○	○	○
I have different social media profiles depending on the type of social media I use (16)	○	○	○	○	○

Q12.3 How much do you agree/disagree with the following statements?

	Strongly disagree (1)	2 (2)	3 (3)	4 (4)	Strongly agree (5)
I find seeing other people's health experiences on social media beneficial to my own health experience (1)	○	○	○	○	○
I can access expert health advice on social media without attending a clinic (2)	○	○	○	○	○
The internet is a good way of finding other people who are experiencing similar health problems (3)	○	○	○	○	○
It can be helpful to see other people's health-related experiences on the internet (4)	○	○	○	○	○

End of Block: 5. Online and social media behaviour

Start of Block: End of survey

Q13.1 Thank you for taking the time to complete this survey.

If would like further information or assistance you can contact one of the researchers:

Tracy McCaffrey: tracy.mccaffrey@monash.edu
Eva Jenkins: eljen4@student.monash.edu
Jasmina Ilicic: jasmina.ilicic@monash.edu
Annika Molenaar: annika.molenaar@monash.edu

Or one of the following services:
- Monash Nutrition Clinic for further information on your diet:
https://www.monash.edu/medicine/base/about/clinics/nutrition-clinic
- Monash counselling services:
https://www.monash.edu/health/counselling
- Lifeline phone line: 13 11 14
- Butterfly foundation phone line: 1800 33 4673

Please click the **SUBMIT SURVEY** button below to exit the survey.

End of Block: End of survey
